# dsRNase1 contribution to dsRNA degradation activity in the Sf9 cells conditioned medium

**DOI:** 10.3389/finsc.2023.1118775

**Published:** 2023-01-24

**Authors:** Jinmo Koo, Subba Reddy Palli

**Affiliations:** Department of Entomology, College of Agriculture, University of Kentucky, Lexington, KY, United States

**Keywords:** *Spodoptera frugiperda*, nuclease, dsRNA stability, RNAi, fall armyworm

## Abstract

RNA interference (RNAi) is inefficient in lepidopteran insects, including *Spodoptera frugiperda*. RNase activity in the lumen and hemocoel is known to contribute to low RNAi efficiency in these insects. Conditioned medium from Sf9 cells developed from ovaries of *S. frugiperda* shows high dsRNA degradation activity. But the enzymes responsible for this activity have not been identified. The nuclease genes that are highly expressed in Sf9 cells, REase, RNaseT2, and dsRNase1, were identified. Knockdown of dsRNase1 in Sf9 cells resulted in a reduction of dsRNA degradation activity in the Sf9 cells conditioned medium. Knockdown of dsRNase1 also increased RNAi efficiency in Sf9 cells. The results from these studies identified a major player in dsRNA degradation activity in the Sf9 cells conditioned medium. We also describe an efficient system that can be used to identify other genes responsible for dsRNA degradation and RNAi efficiency in Sf9 cells.

## 1 Introduction

RNA interference (RNAi) efficiency varies among insects (1). High nuclease activity in lepidopteran insects is one of the major factors responsible for low RNAi efficiency in these insects (2). dsRNase, which belongs to the non-specific endonuclease family, is known to be the major nuclease that degrade dsRNA in insects (1, 3). Most of the time, the function of dsRNase is highlighted in the midgut (4, 5), since its expression is normally high in the midgut (6). dsRNase is usually active in alkaline pH (3, 7). Lepidopteran insects have an alkaline pH in the lumen (8), which makes a favorable environment for dsRNases. However, the study of dsRNases in other tissues and their effect on RNAi efficiency are limited. Insect genomes normally have three to five dsRNase genes (9, 10). Although most dsRNase genes are known to be highly expressed in the midgut, some genes are also expressed in other tissues. For example, In *Spodoptera litura*, dsRNase1, 2, and 4 are highly expressed in the midgut. But dsRNase3 is expressed in the silk gland, fat body, and integument, and dsRNase2 is expressed in the head (11). This tissue-specific dsRNase expression may affect tissue-specific or overall RNAi efficiency. Therefore, the influence of dsRNase on the RNAi efficiency of various tissues and cell lines derived from these tissues can provide valuable information.

Sf9 cells, derived from *Spodoptera frugiperda* ovarian tissue, are refractory to RNAi (12). Various dsRNA delivery technologies have been explored to improve RNAi in *S. frugiperda*. Sf9 cells are frequently used as an *in vitro* system to check RNAi efficiency before moving on to *in vivo* assays. Although Sf9 cells don’t represent all the target tissues present in *S. frugiperda*, it has served great system for preliminary studies to test different dsRNA delivery methods (13–15). Some of these delivery methods were transferable to *in vivo* assays (16). Sf9 cells also has served as an important tool to study mechanism of RNAi and to discover factors that affect RNAi efficiency (12, 17, 18). Therefore, Sf9 cells are an important system for developing RNAi technologies, and it is important to further study factors that affect RNAi efficiency in this cell line. Conditioned medium from Sf9 cells have strong nuclease activity that degrades dsRNA (13–15). However, proteins responsible for this nuclease activity in the conditioned medium is unknown.

In this study, we identified nucleases that are highly expressed in Sf9 cells. These nucleases include dsRNases, REase, and RNaseT2. REase, first identified in Asian corn borer, is known to be a lepidopteran-specific nuclease that suppresses RNAi (19). RNaseT2 is an endonuclease predicted to be responsible for rRNA and tRNA recycling and share its function with RNaseA in vertebrates (20). Sf9_SID1_Luciferase cell line expresses *Caenorhabditis elegans* Systemic RNAi defective (SID1) protein. SID1 is a dsRNA channel protein that is responsible for the inter-cellular systemic spread of dsRNA in *C. elegans* (21). It is reported that expressing *C. elegans* SID1 in Sf9 cells confers efficient RNAi response (22). We used the Sf9_SID1_Luciferase cell line to knockdown candidate nucleases to check if dsRNA stability in the conditioned medium can be improved. Since this cell line also expresses luciferase protein, we used luciferase assay to check whether knocking down these nucleases can further improve RNAi efficiency.

## 2 Material and methods

### 2.1 Cell line

Sf9_SID1_Luciferase cell line was generated as described previously (22). Sf-900 II SFM medium (Thermo Fisher Scientific Inc., USA) was used to maintain this cell line.

### 2.2 RNA isolation and cDNA synthesis

RNA was isolated using Trizol (Molecular Research Center Inc., Cincinnati, OH). To synthesize cDNA, M‐MLV Reverse Transcriptase (Invitrogen™) kit was used. One microgram of total RNA, dNTP, random primer, and oligo primer were first mixed in 13 µl volume. The mixture was incubated at 65°C for 5 min. Then, 5X buffer, DTT, and reverse transcriptase were added to make up the volume to 20 µl. The mixture was incubated at 37°C for 1 hour, followed by 70°C for 15 minutes for enzyme inactivation.

### 2.3 qRT-PCR

Quantitative real-time PCR (qRT-PCR) reaction (10 μL final volume) contained 5 μL of 2X SYBR Mix (BioRad, USA), 2 μL of 5-fold diluted cDNA, 2.2 μL of nuclease-free water, and 0.4 μL each of 10 µM forward and reverse gene-specific primers. An initial incubation of 95°C for 3 min, followed by 40 cycles of 95°C for 5s, 60°C for 60s settings, were used. A melt curve was generated at the end of run to confirm a single peak and rule out the possibility of primer-dimer and non-specific product formation. qRT-PCR reactions were performed in Applied Biosystems Step One Plus™ Real-Time PCR System. The relative mRNA levels were calculated as a fold change over the mRNA levels of the reference gene, 28S rRNA (ΔCt method). Our lab has been using 28S rRNA for Sf9 cells for multiple publications (13, 15, 17, 23). 28S rRNA CT-value is stable with various dsRNA and hormone treatment. Primers used for qRT-PCR reactions are listed in **Table S1**. Primer efficiency is listed in **Table S2**. Cells seeded in different wells were processed as individual samples. To maintain stability of the reference gene, only samples with CT-value difference less than one for 28S rRNA were selected for further analysis.

### 2.4 dsRNA synthesis

Total RNA isolated from Sf9 cells was used to synthesize cDNA. Using cDNA as a template, fragments of the target gene with 5’ T7 promoter sequence were amplified with PCR using designed primers (**Table S1**). PCR amplifications were conducted in 50 µl reactions containing 5 µM concentration of each primer, 25 µl of 2x Taq premix (Promega) and 5 µl of synthesized cDNA. PCR conditions were 95°C for 3 min, followed by 35 cycles of 95°C for 30s, 57°C for 30s and 68°C for 1 min, finishing with an extension step at 68°C for 5 min. PCR products were purified using the QIAquick PCR purification kit (QIAGEN). The purified PCR products were used as templates to synthesize dsRNAs using Megascript T7 RNA synthesis kit (Life Technologies, Carlsbad, CA).

### 2.5 dsRNA stability assay

To obtain conditioned medium from cells depleted with target nuclease, Sf9_SID1_Luciferase cells seeded in 48 well plates were first treated with 1 µg dsRNA targeting different nucleases. Untreated cells or cells treated with dsRNA targeting green fluorescence protein (dsGFP) was used as control. Cells were incubated 2 days to allow knockdown of target nuclease genes. After, fresh medium was replaced and allowed cells to secrete proteins into the medium for 2 days. Medium was collected and processed by taking supernatant after centrifuging at 5,000 g for 10 minutes to remove cell debris. One µg dsGFP was incubated with 19 µl of conditioned medium at 28°C for various lengths of time and the products were run on 1% agarose gels.

To assess the intracellular dsRNA stability, cell lysate was obtained. Sf9_SID1_Luciferase cells seeded in 48 well plates were treated with 1 µg dsRNA targeting different nucleases. After two days of incubation, the cells were washed with 1x PBS buffer two times. After washing, the cells were harvested using 1x PBS buffer. Harvested cells were lysed with sonication. Cell debris was removed by centrifugation of lysate at 5,000 g for 10 minutes.

### 2.6 Luciferase assay

Sf9_SID1_Luciferase cells seeded in 48 well plates were treated with 1 µg dsRNA targeting different nucleases. After two days of incubation, fresh medium was replaced containing 0.3 ng of either dsGFP or dsLuc and incubated for two additional days. The cells were washed with 1x PBS buffer and lysed with 200 μl of lysis buffer for 10 min at room temperature. Then 20 μl of cell lysate was used for luciferase activity assay. The luciferase activity was measured using SpectraMax i3x (Molecular Devices). Bradford assay was performed to make sure that all the cell lysates used for luciferase assay had equivalent protein concentrations.

### 2.7 Statistical analysis

SMART (http://smart.embl-heidelberg.de/) was used to find significant conserved domains and signal peptides from translated amino acid sequence of nucleases. To compare the means between two groups (control and treatment), a two-tailed t-test was used. Three biological replicates of cells seeded in different wells were used for each treatment (N=3). To verify normal distribution of the replicates, Shapiro-Wilk test was performed using a tool from Statistics Kingdom website (https://www.statskingdom.com/shapiro-wilk-test-calculator.html). F-test with p value of 0.05 was used to determine equal variance among the treatment. t-test and F-test was done in Excel Version 2211 (Microsoft).

## 3 Results

### 3.1 Expression of nuclease genes in Sf9_SID1_Luciferase cells

**Figure 1:**
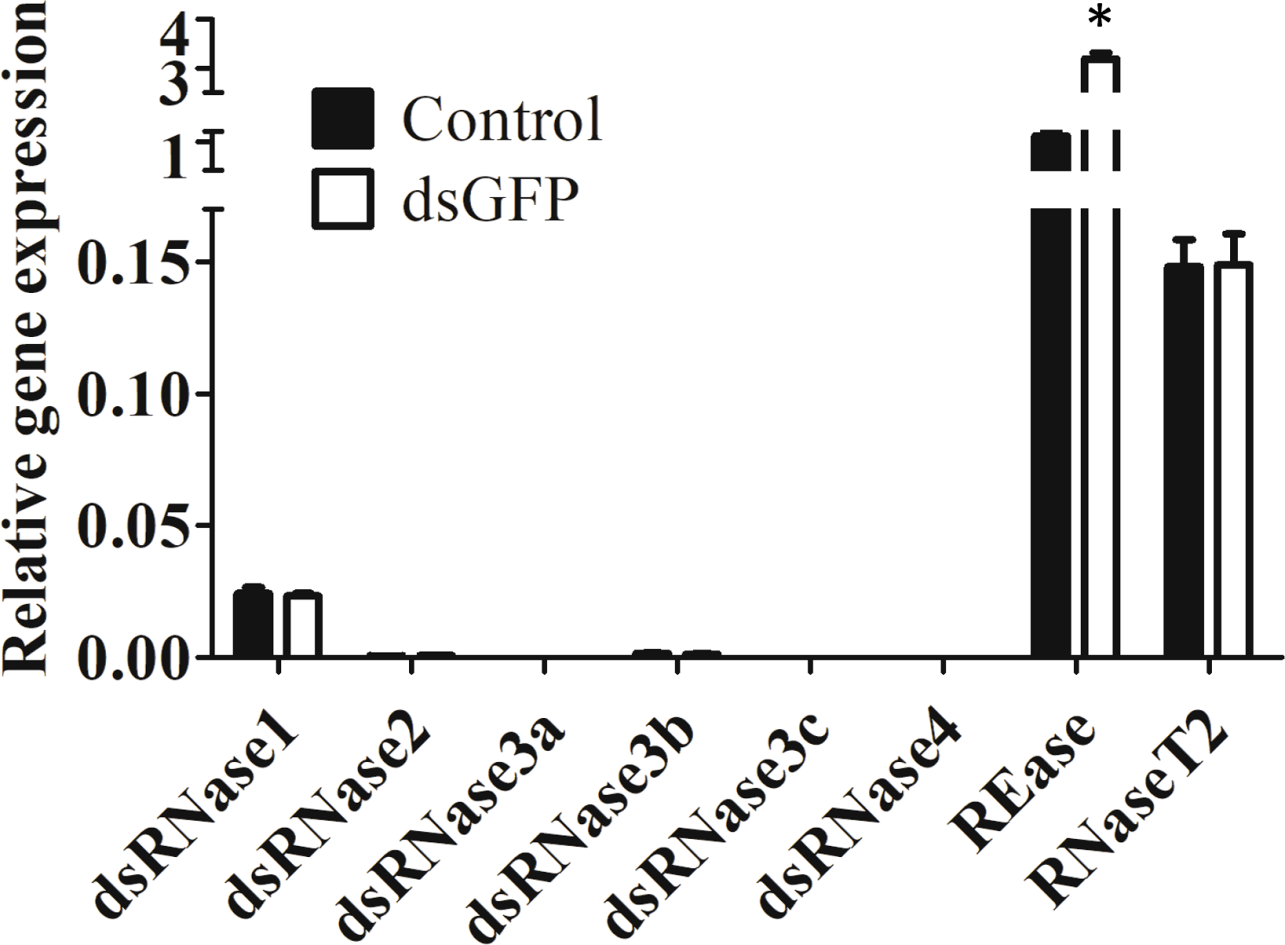
Expression of nucleases in Sf9_SID1_Luciferase cells. Sf9_SID1_Luciferase cells seeded in 48 well plates were treated with either water control or 1 µg dsGFP. After three days of incubation, the cells were harvested, total RNA was isolated and used in qRT-PCR to quantify relative mRNA levels of target genes. Mean ± SE (N = 3) are shown. ‘*’ denotes significant differences in the mRNA levels of target genes between control and dsGFP treatments (P < 0.05).

Expression of eight nuclease genes in Sf9_SID1_Luciferase cells was determined. Six dsRNase genes were identified by BLAST searches of *S. litura* dsRNase nucleotide sequences (11) against *S. frugiperda* transcriptome (**Figure S1**). REase is a lepidopteran-specific nuclease belonging to PIN family (19). RNaseT2 catalyzes endonucleolytic RNA cleavage (20). REase showed the highest expression followed by RNaseT2 and dsRNase1 in Sf9_SID1_Luciferase cells (**Figure 1**). Expression profiles of these genes in Sf9 cells also showed a similar pattern (**Figure S2**). dsGFP treatment of these cells increased REase mRNA levels significantly, but the expression of other genes was not affected by this treatment (**Figure 1**).

### 3.2 Knockdown of dsRNase1 improved dsRNA stability of Sf9_SID1_Luciferase cell conditioned medium

**Figure 2:**
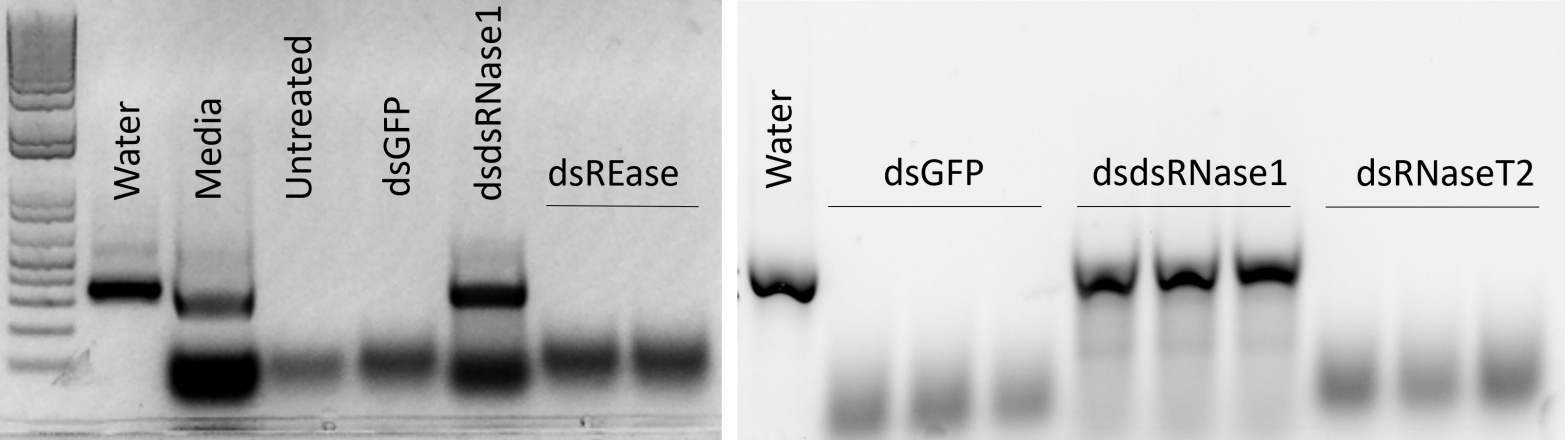
Knockdown of dsRNase1 improved dsRNA stability in conditioned medium from Sf9_SID1_Luciferase cells. Sf9_SID1_Luciferase cells seeded in 48 well plates were treated with 1 µg dsRNA targeting different nuclease genes. After two days of incubation, fresh medium was replaced, and incubated for two additional days. The medium was collected and used in dsRNA degradation assay. One µg dsGFP was incubated with 19 µl of the conditioned medium at 28°C for 5 hours and the products were run on 1% agarose gels. dsGFP incubated with water (Water), Sf-900 medium (Medium), conditioned medium from untreated cells (Untreated), conditioned medium from dsRNA treated cells targeting GFP (dsGFP), dsRNase1 (dsdsRNase1), REase (dsREase), and RNaseT2 (dsRNaseT2).

**Figure 3:**
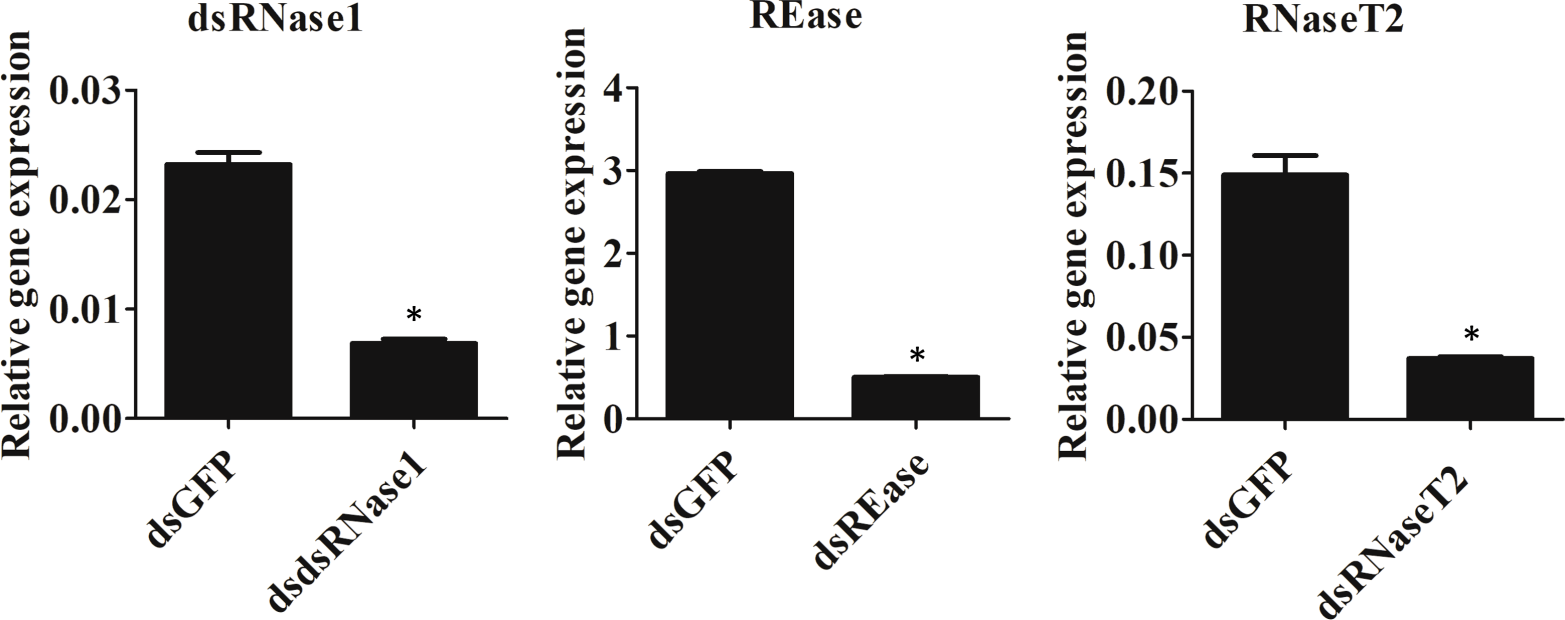
Knockdown efficiency of nuclease genes. Sf9_SID1_Luciferase cells seeded in 48 well plates were treated with 1 µg dsRNA targeting different nucleases. After two days of incubation, cells were harvested, and the total RNA was isolated and used in qRT-PCR. Mean ± SE (N = 3) are shown. ‘*’ denotes significant differences in the expression levels of target genes between control and treatments (P < 0.05).

We focused on the three nucleases, REase, RNaseT2, and dsRNase1, since they were the top three expressed nucleases tested. To determine which genes are responsible for dsRNA degradation activity in conditioned medium, each gene was silenced by treating the cells with targeting dsRNA. The conditioned medium collected from the dsRNA-treated cells was used for dsRNA stability assay. Conditioned medium taken from dsRNase1 knockdown cells showed substantially lower dsRNA degradation compared that in conditioned medium from untreated and dsGFP treated cells. In contrast, REase and RNaseT2 knockdown didn’t affect dsRNA degradation activity in the conditioned medium (**Figure 2**). When we incubated dsRNA in the conditioned medium for different lengths of time, dsRNA was stable up to 30 hr when dsRNase1 was knocked down (**Figure S3**). It is important to note that all six dsRNases and RNaseT2 have signal peptides in their N-terminal region, but a signal peptide is absent in REase (**Figure S1**). One can predict that REase may be responsible for dsRNA stability inside the cell. To test this hypothesis, we performed dsRNA stability assay with cell lysate. However, we couldn’t find difference in dsRNA degradation ability of cell lysates collected from dsGFP and dsREase treated cells (**Figure S4**). Knockdown of dsRNase1, RNaseT2 and REase gene expression was confirmed by qRT-PCR (**Figure 3**). Expression of other nuclease genes that are not targeted was not affected, confirming the specificity of the designed dsRNA (**Figure S5**).

### 3.3 Knockdown of dsRNase1 improved RNAi efficiency in Sf9_SID1_Luciferase cells

**Figure 4:**
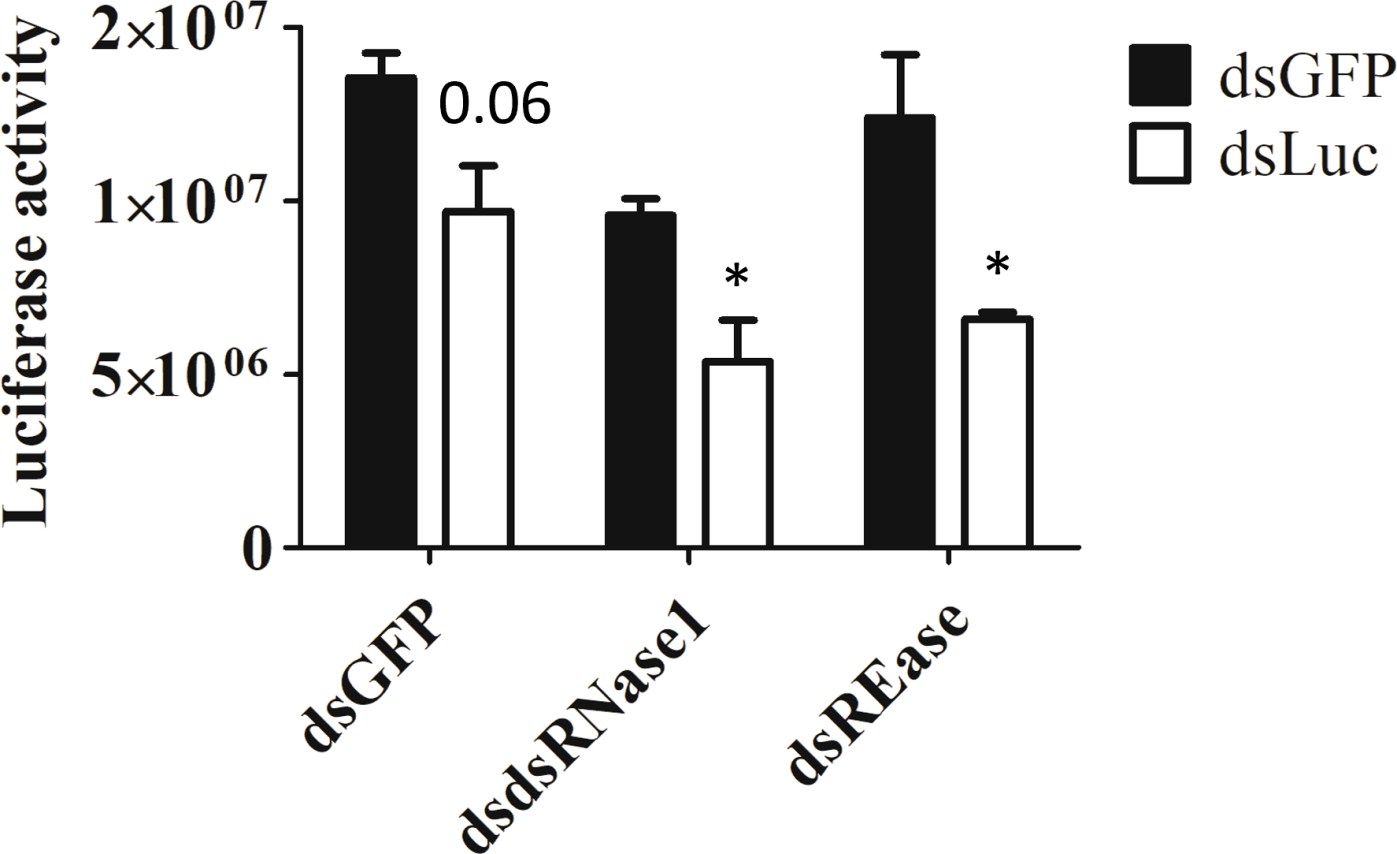
Knockdown of dsRNase1 and REase enhances RNAi efficiency. Sf9_SID1_Luciferase cells seeded in 48 well plates were treated with 1 µg dsRNA targeting different nucleases. After two days of incubation, 0.3 ng of either dsGFP or dsLuc were added to the medium and cultured for additional two days. Cells were collected and lysates were prepared and used for luciferase assay. Luciferase activity was normalized with the protein concentration measured by Bradford assay (**Figure S6**). Mean ± SE (N = 4) is shown. ‘*’ denotes significant differences in the luciferase activity between dsGFP and dsLuc treatment (P < 0.05). P-value > 0.05 was indicated as actual value.

Now we know that dsRNase1 is a major contributor to dsRNA degradation activity in the medium, we checked whether knocking down dsRNase1 improves RNAi in Sf9_SID1_Luciferase cells. RNAi of RNAi approach was used. When dsRNase1 was knocked down, the second dsRNA, dsLuc showed increased knockdown efficiency (28 → 44%) (**Figure 4**). Knocking down REase also improved RNAi efficiency (28 → 46%). Bradford assay was performed to make sure that all the cell lysates analyzed in luciferase assay had equivalent protein concentrations (**Figure S6**).

## 4 Discussion

In Sf9 cells, dsRNase1 was highly expressed among six dsRNase genes. Indeed, dsRNase1 seems to have a dominant role in dsRNA degradation activity in the conditioned medium. However, degradation of dsRNA was detected after longer incubation (50 hr) in a conditioned medium from dsRNase1 knockdown cells (**Figure S3**). This activity may be due to incomplete knockdown of dsRNase1 gene, other dsRNases like dsRNase3b, or other nucleases not identified yet. Further studies are needed to distinguish among these possibilities. Knockdown of dsRNase1 substantially increased dsRNA stability in conditioned medium. However, the increase in RNAi efficiency was moderate (**Figure 4**). There are other additional steps, such as cellular uptake and intracellular localization, before dsRNA reaches RNAi machinery proteins (24). Further investigations are needed to check whether increased dsRNA stability in conditioned medium leads to increased uptake of dsRNA into Sf9 cells. Intracellular localization of dsRNA is known to be another major factor responsible for low RNAi efficiency in *S. frugiperda* and Sf9 cell (12, 17). This may be the reason why we had limited improvement of RNAi efficiency in Sf9 cells even after we improve extracellular dsRNA stability. dsRNase1 knockdown itself decreased the luciferase activity slightly compared to dsGFP treated cells. It is possible that dsRNase1 may have pleiotropic effects. dsRNase1 knockdown cells grew normally and had similar amount of protein produced compared to dsGFP treated cells (**Figure S6**). Further research is needed to check whether dsRNase have additional functions in cells other than nuclease activity. Additional research is also required to check whether dsRNase1 is expressed in midgut or in other tissues. Wherever it is expressed, it looks clear that dsRNase1 is a functional nuclease that degrade extracellular dsRNA.

In Sf9_SID1_Luciferase cells, dsGFP treatment didn’t upregulate any of dsRNase genes (**Figure 1**). Few reports observed upregulation of dsRNase genes by dsRNA treatment (25, 26). In *Bombyx mori*, upregulation of dsRNase was observed only by injection of dsGFP but not feeding (25). dsRNA delivery method may affect upregulation of dsRNase genes. In *Ostrinia nubilalis*, dsRNase4 but not dsRNase2 was upregulated by dsGFP injection. Different dsRNase genes may response differently to exogenous dsRNA treatment. REase gene expression was highly upregulated by dsGFP treatment in Sf9_SID1_Luciferase cells (**Figure 1**). This result is consistent with the previous observation in *Ostrinia furnacalis* (19), and *Ostrinia nubilalis* (26).

The exact cellular localization of REase protein is not investigated yet. Unlike dsRNases, REase peptide sequence does not contain a signal peptide (**Figure S1**), so it may not be secreted out of the cells and retained in the cytoplasm and affect dsRNA stability in the cytosol. Indeed, conditioned medium from REase-depleted cells showed dsRNA degradation activity comparable to untreated cells (**Figure 2**). High expression in the cells but lack of evidence of activity in conditioned medium suggests that REase is either retained in the cytoplasm or if it is secreted into the medium or released after cell lysis, the environment (Sf-900 medium) is not conducive for activity of this enzyme. The lack of signal peptide supports retention in the cytoplasm hypothesis. We tested cell lysate to see if the nuclease activity of REase is retained in the cells. However, we were not able to detect any changes in dsRNA stability between lysates from dsGFP and dsREase treated cells (**Figure S4**). Guan et al. (19), reported that purified REase enzyme activity was quite low, and three days of incubation period was required for dsRNA degradation. They also proposed that in addition to nuclease activity, REase have competitive relationship with Dicer-2, reducing unique and total reads of small RNAs digested from Dicer protein (19). Further research is needed to understand the mechanism and which specific steps of RNAi pathway are affected by REase. Activities of enzyme are modulated by intracellular milieu (27). It is possible that intracellular nuclease activity may be affected in the lysate condition. Recently, dsRNA was reported to be stable in the presence of lysate from Hi5 cell line of *Trichoplusia ni* (28). Knockdown of REase increased RNAi efficiency moderately (**Figure 4**). Consistent with our result, REase knockout in Asian corn borer had limited improvement in RNAi efficiency (29).

Although there is no report of RNaseT2 activity in dsRNA degradation, its basal property (contains a signal peptide and has endonuclease function) made it a possible candidate for such function (**Figure S1**). Our result showed that RNaseT2 is not responsible for dsRNA degradation activity in the conditioned medium of Sf9 cells. Although the data did not support our hypothesis, similar approaches should be further explored to find other nuclease candidates responsible for dsRNA stability and RNAi efficiency.

Sf9_SID1_Luciferase cells have efficient RNAi (22). Also, transfection and overexpression are amenable in Sf9 cells. Therefore, Sf9_SID1_Luciferase is a versatile system to knockdown or overexpress any target genes including nucleases. Change in dsRNA stability either in conditioned media or whole cell lysate can be measured to quantify dsRNA degradation activity and distinguish active location of the candidate nuclease. Sf9_SID1_Luciferase cells also allows us to check RNAi efficiency using luciferase assay. We believe that this system provides a convenient tool for preliminary screening of potential nucleases in *S. frugiperda*.

## Data availability statement

The original contributions presented in the study are included in the article/**Supplementary Material**. Further inquiries can be directed to the corresponding author.

## Author contributions

JK and SP designed research. JK performed research. JK and SP analyzed data. JK and SP wrote the paper. All authors contributed to the article and approved the submitted version.
