# Supplementary material for: dsRNase1 contribution to dsRNA degradation activity in the Sf9 cells conditioned medium
Source: Front Insect Sci. 2023 Jan 24;3:1118775. doi: 10.3389/finsc.2023.1118775 (PMC10926405; doi:10.3389/finsc.2023.1118775)
Supplement: Supplementary file 1 [file DataSheet_1.pdf]

Supplementary information

dsRNase1 contribution to dsRNA degradation activity in the Sf9 cells conditioned medium

Jinmo Koo[a] and Subba Reddy Palli[a]*

[a] Department of Entomology, College of Agriculture, University of Kentucky, Lexington, KY 40546, USA

*Corresponding author: rpalli@uky.edu

Figures

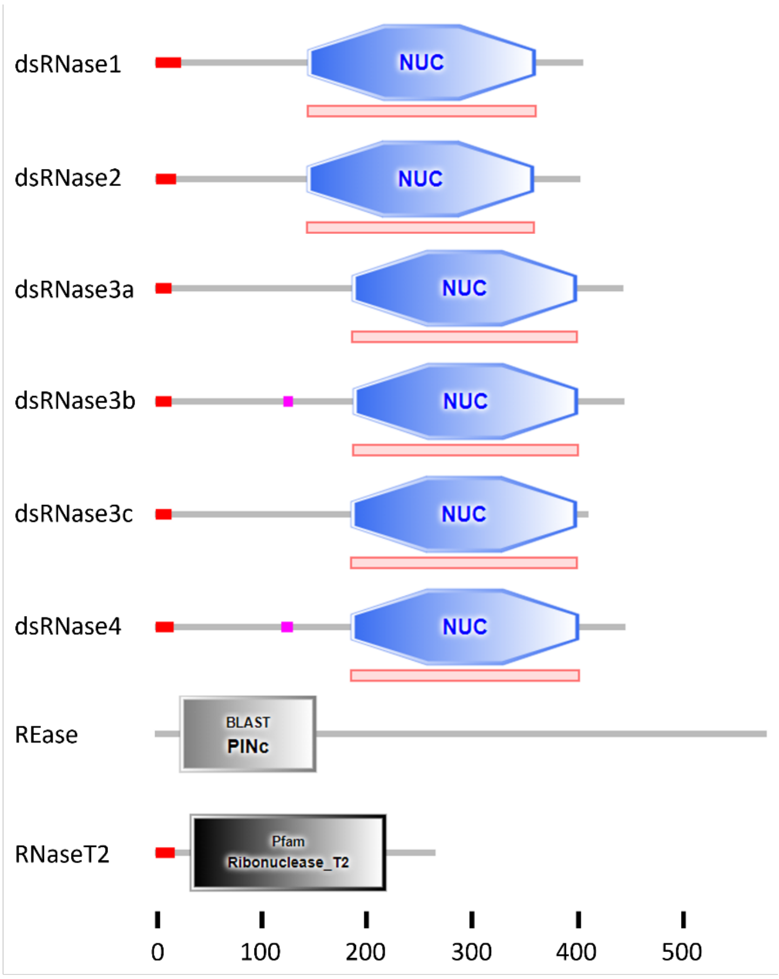

Figure S1. Characteristic of nucleases. Conserved domains and signal peptides are indicated for each nuclease. Accession numbers of genes can be found in Table S1. NUC, DNA/RNA non-specific endonuclease domain. PINc, large family of predicted nucleotide-binding domain. Ribonuclease_T2, Ribonuclease_T2 domain. Red box, signal peptide. Pink box, low complexity region. Numbers at the bottom indicate amino acid residues.

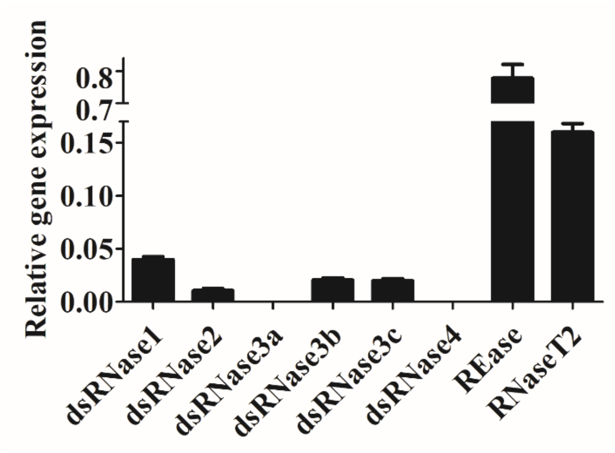

Figure S2. Expression of nucleases in Sf9 cells. Total RNA was isolated from Sf9 cells and qRT-PCR was used to determine mRNA levels of nuclease genes. Mean ± SE (N = 3) are shown.

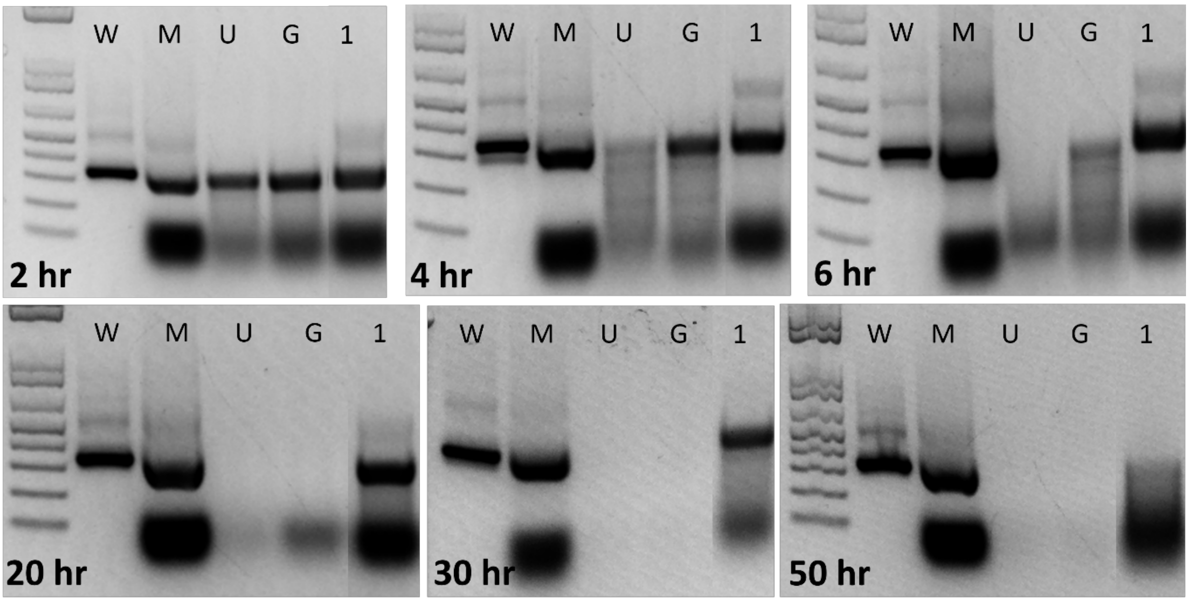

Figure S3. dsRNA is stable up to 30 hr in conditioned medium from dsRNase1 knockdown cells. Sf9_SID1_Luciferase cells seeded in 48 well plates were treated with 1 µg dsRNA. After two days of incubation, the medium was replaced with fresh medium, allowing cells to secrete nucleases into the replaced medium for two additional days. Conditioned medium was collected and incubated with 1 µg dsGFP in 28ºC for different lengths of time (2, 4, 6, 20, 30, 50 hr). The products were run on 1% agarose gels. dsGFP incubated with water (W), Sf-900 medium (M), conditioned medium from untreated cells (U) or conditioned medium from cells treated with dsRNA targeting GFP (G) and dsRNase1 (1).

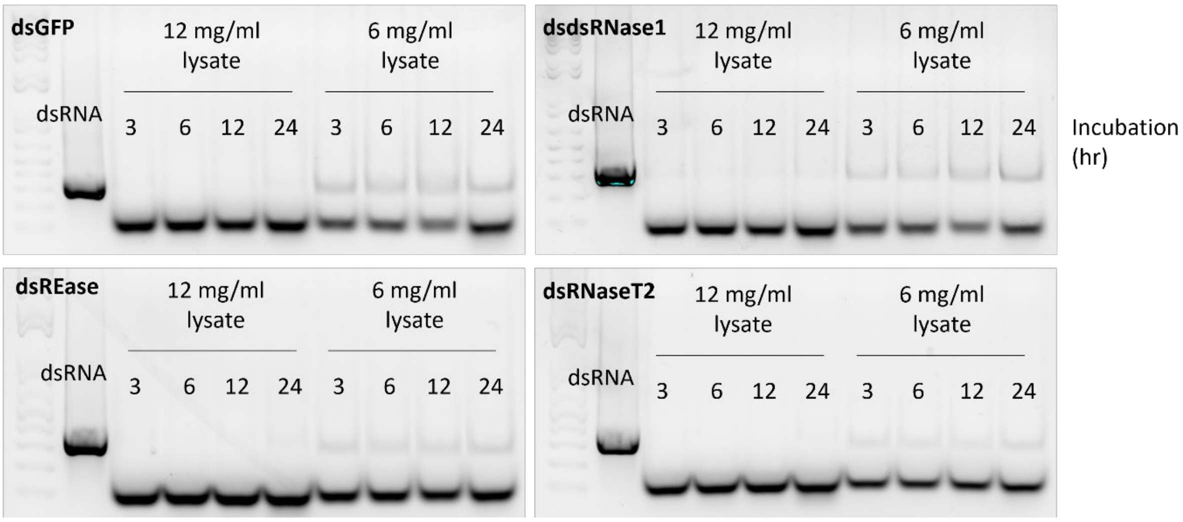

Figure S4. dsRNA stability in cell lysate. Sf9_SID1_Luciferase cells seeded in 48 well plates were treated with 1 µg dsRNA targeting different nucleases (dsRNase1, REase, and RNaseT2). After two days of incubation, cells were harvested and lysates were prepared. One µg dsGFP was mixed with 19 µl of cell lysate with different concentrations (6 and 12 mg/ml) and incubated at 28°C for different lengths of time (3, 6, 12, and 24 hr). The products were run on 1% agarose gels.

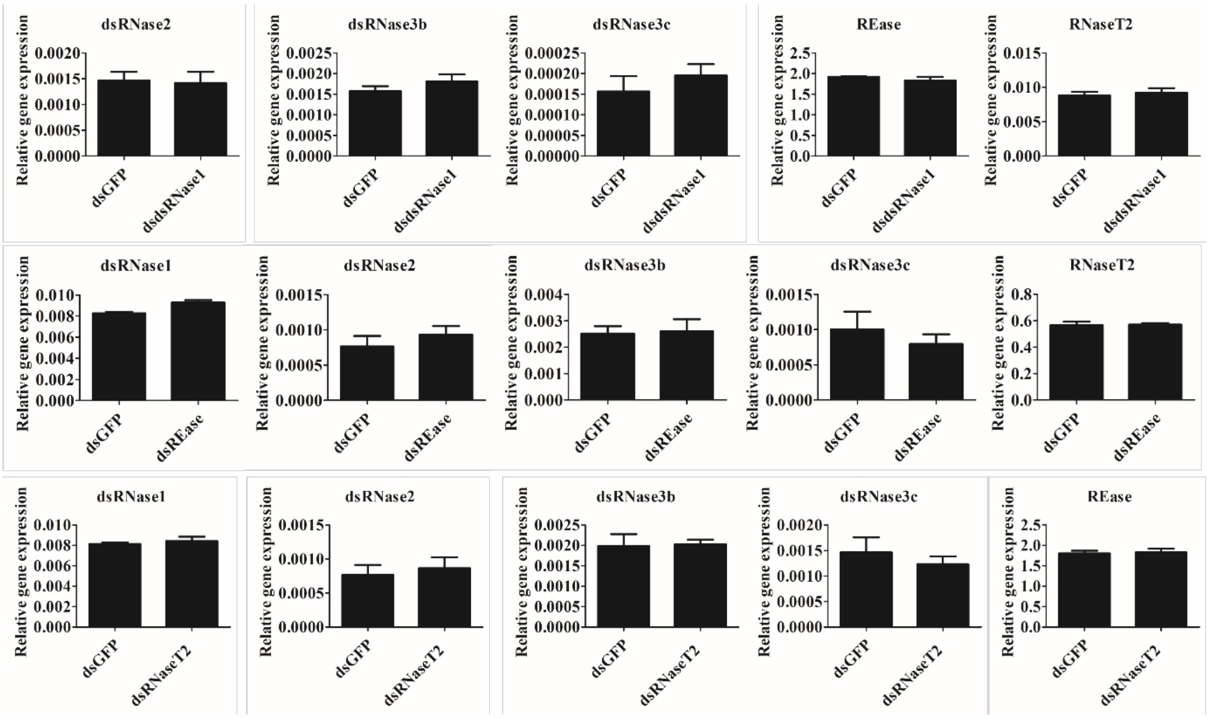

Figure S5. Specificity of dsRNA targeting dsRNase1, REase, and RNaseT2. Sf9_SID1_Luciferase cells seeded in 48 well plates were treated with 1 µg dsRNA targeting different nucleases (dsRNase1, REase, and RNaseT2). After two days of incubation, cells were harvested, and the total RNA was isolated and used in qRT-PCR. Mean ± SE (N = 3) are shown. Expression of dsRNase3a and 4 were not included since expression of those genes was not

detected in Sf9_SID1_Luciferase cells.

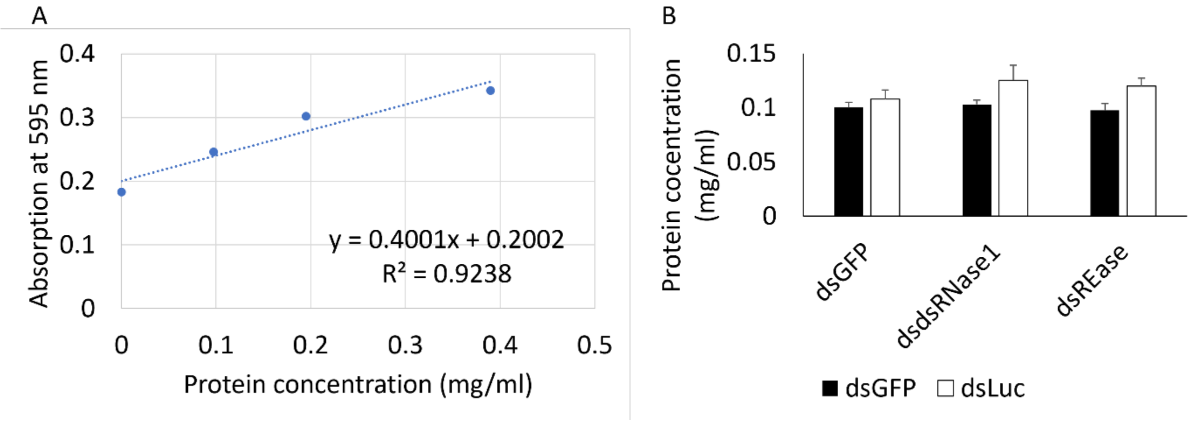

Figure S6. Bradford assay. Protein concentration was quantified using Bradford assay for cell lysate used in luciferase assay (Figure 4). A. standard curve was generated using serial dilutions of Bovine serum albumin. B. protein concentrations of each samples calculated using the standard curve.

Tables

Table S1. Primer information. T7 promoter sequnce is included in 5' region of all dsRNA primers.

Primers	Sequence (5'-3')	Purpose	Tm (°C)	Product size (bp)	Accession
28SrRNA_qRTF	CTGCTTACAGAGACGAGGTTAAG	qPCR	63	103	AY046536.1
28SrRNA_qRTR	GGGTAGTAGTCCAGACCAGAAT	qPCR	64		
dsRNase1_qRTF	CGTCGTGTCCATGAGTTAGG	qPCR	63	91	XM_035598562.1
dsRNase1_qRTR	GATAAAGGAGGTCGGATGAAGG	qPCR	63		
dsRNase2_qRTF	GTGATACCGGTGCCTCTATTT	qPCR	63	108	XM_035587097.2
dsRNase2_qRTR	TTATGTGTCTTCAACTGAGGCA	qPCR	63		
dsRNase3a_qRTF	CACGTGTTCAGCACACTCTA	qPCR	63	88	XM_035575402.1
dsRNase3a_qRTR	TTATCCGTGTCGGCTTCATC	qPCR	63		
dsRNase3b_qRTF	GTGGCTGTCAACACCATGTA	qPCR	63	99	XM_035575401.1
dsRNase3b_qRTR	AAATACATCACCACCCACCAG	qPCR	63		
dsRNase3c_qRTF	GCCAATGCGTCTGTTGAAG	qPCR	63	116	XM_035575404.1
dsRNase3c_qRTR	CTGGCAGCCAAGTATCTGT	qPCR	63		
dsRNase4_qRT1F	GGACGTATTCTACCCGCTATAC	qPCR	63	102	XM_035575400.2
dsRNase4_qRT1R	CATAACGCTGTCCACCAAAC	qPCR	62		
dsRNase4_qRT2F	CACATCCAACTCACGAGGCT	qPCR	65	137	XM_035575400.2
dsRNase4_qRT2R	AACTTCGAGGCGACGTTCAT	qPCR	65		
REase_qRTF	CGAGAATGGTTGGGAGGATATAG	qPCR	63	105	XM_035594883.2
REase_qRTR	ACGCTGTCATCTGGTAGTTTAG	qPCR	63		
RNaseT2_qRTF	TAGAGCTCCGAGTTTGCTTTG	qPCR	63	127	XM_035578184.2
RNaseT2_qRTR	GGAGGTAATGCAGTGGGATAAA	qPCR	63		
Luciferase_dsF	TATCCGCTGGAAGATGGAAC	dsRNA	62	355	MK484107.1
Luciferase_dsR	ACCCCTTTTTGGAAACGAAC	dsRNA	62		
GFP_dsF	CGATGCCACCTACGGCAA	dsRNA	65	248	MN623123.1
GFP_dsR	TGTCGCCCTCGAACTTCA	dsRNA	64		
dsRNase1_dsF	ACATCCGCTGTAACTCCCAC	dsRNA	65	553	XM_035598562.1
dsRNase1_dsR	GTCATCATTCCATGGTTCCC	dsRNA	62		
REase_dsF	CTCCCAACCATTCTCGAATACA	dsRNA	63	473	XM_035594883.2
REase_dsR	AGCACGAGGAGCAAGAAATA	dsRNA	62		
RNaseT2_dsF	CTCAACAATGGCCTAGCTCC	dsRNA	64	451	XM_035578184.2
RNaseT2_dsR	ACACCGATCTTGGAAGCAAC	dsRNA	64		

Table S2. qPCR primer efficiency. CT-values from diluted cDNA (1, 0.1, 0.01) were ploted. Slope from the plot was used to calculate primer efficiency using a tool from ThermoFisher website (https://www.thermofisher.com/us/en/home/brands/thermo-scientific/molecular-biology/molecular-biology-learning-center/molecular-biology-resource-library/thermo-scientific-web-tools/qpcr-efficiency-calculator.html). NTC, non target control. Undeter, CT-value not determined due to no amplification. NA, not available. P1, primer1. P2, primer2.

		28SrRNA	dsRNase1	dsRNase2	dsRNase3a	dsRNase3b	dsRNase3c	dsRNase4_P1	dsRNase4_P2	REase	RNaseT2
CT-values	1 dilution	21.29	28.37	30.74	24.32	29.75	31.31	Undeter	Undeter	20.64	22.17
	0.1 dilution	24.87	31.47	34.65	28.15	33.35	34.04	Undeter	Undeter	23.75	25.53
	0.01 dilution	27.89	35.07	37.42	31.28	35.87	37.35	Undeter	Undeter	27.27	28.83
	NTC	Undeter	Undeter	Undeter	Undeter	Undeter	Undeter	Undeter	Undeter	Undeter	38.07
Slope		-3.30	-3.35	-3.34	-3.48	-3.06	-3.02	NA	NA	-3.31	-3.33
primer efficiency (%)		100.9	98.8	99.3	94.0	112.3	114.1	NA	NA	100.3	99.6
